# Living Chinese Herbal Scaffolds from Microfluidic Bioprinting for Wound Healing

**DOI:** 10.34133/research.0138

**Published:** 2023-05-09

**Authors:** Xiaocheng Wang, Jinxuan Jia, Mengying Niu, Wenzhao Li, Yuanjin Zhao

**Affiliations:** ^1^ Department of Rheumatology and Immunology, Nanjing Drum Tower Hospital, School of Biological Science and Medical Engineering, Southeast University, Nanjing 210096, China.; ^2^ Oujiang Laboratory (Zhejiang Lab for Regenerative Medicine, Vision and Brain Health), Wenzhou Institute, University of Chinese Academy of Sciences, Wenzhou, Zhejiang 325001, China.; ^3^ State Key Laboratory of Bioelectronics, School of Biological Science and Medical Engineering, Southeast University, Nanjing 210096, China.; ^4^ Chemistry and Biomedicine Innovation Center, Nanjing University, Nanjing 210023, China.

## Abstract

Biological scaffolds have been widely employed in wound healing applications, while their practical efficiency is compromised by insufficient oxygen delivery to the 3-dimensional constructs and inadequate nutrient supply for the long-term healing process. Here, we present an innovative living Chinese herbal scaffold to provide a sustainable oxygen and nutrient supply for promoting wound healing. Through a facile microfluidic bioprinting strategy, a traditional Chinese herbal medicine (*Panax notoginseng* saponins [PNS]) and a living autotrophic microorganism (microalgae *Chlorella pyrenoidosa* [MA]) were successfully encapsulated into the scaffolds. The encapsulated PNS could be gradually released from the scaffolds, which promoted cell adhesion, proliferation, migration, and tube formation in vitro. In addition, benefiting from the photosynthetic oxygenation of the alive MA, the obtained scaffolds would produce sustainable oxygen under light illumination, exerting a protective effect against hypoxia-induced cell death. Based on these features, we have demonstrated through in vivo experiments that these living Chinese herbal scaffolds could efficiently alleviate local hypoxia, enhance angiogenesis, and thereby accelerate wound closure in diabetic mice, indicating their great potential in wound healing and other tissue repair applications.

## Introduction

Cutaneous wound management has been a major healthcare burden worldwide due to the increasing incidence of trauma and pathophysiological conditions [1–3]. Generally, wound healing represents a highly orchestrated dynamic process with 3 overlapping stages including inflammation, proliferation, and tissue remodeling [4–6]. Impaired or non-healing wounds often occur in case of large-scale trauma or disease conditions such as diabetes [7–10]. Thus, numerous wound dressing materials or biological scaffolds have been developed for wound healing applications [11–17]. Particularly, 3-dimensional (3D)-printed scaffolds have attracted considerable attention owing to their distinctive advantages including the controllable geometric shapes, tailorable porous architectures, and flexible compositions [18–21]. These porous scaffolds can not only provide a structural support for cellular migration and tissue ingrowth, but also serve as effective carriers for the delivery of desirable bioactive molecules to the wound beds [12,22]. However, current wound dressings often suffer from their poor loading efficiency, necessitating frequent dressing changes until the wound heals completely [12,23,24]. Although some stimulus-responsive delivery systems would realize on-demand drug release in a short term (several hours or days), they usually hardly maintain the adequate drug levels for the long-term healing process [25,26]. Therefore, it is still highly anticipated to design novel scaffolds that could continuously supply the desirable bioactive molecules to meet the dynamic need throughout the whole wound healing process.

**Fig. 1.**
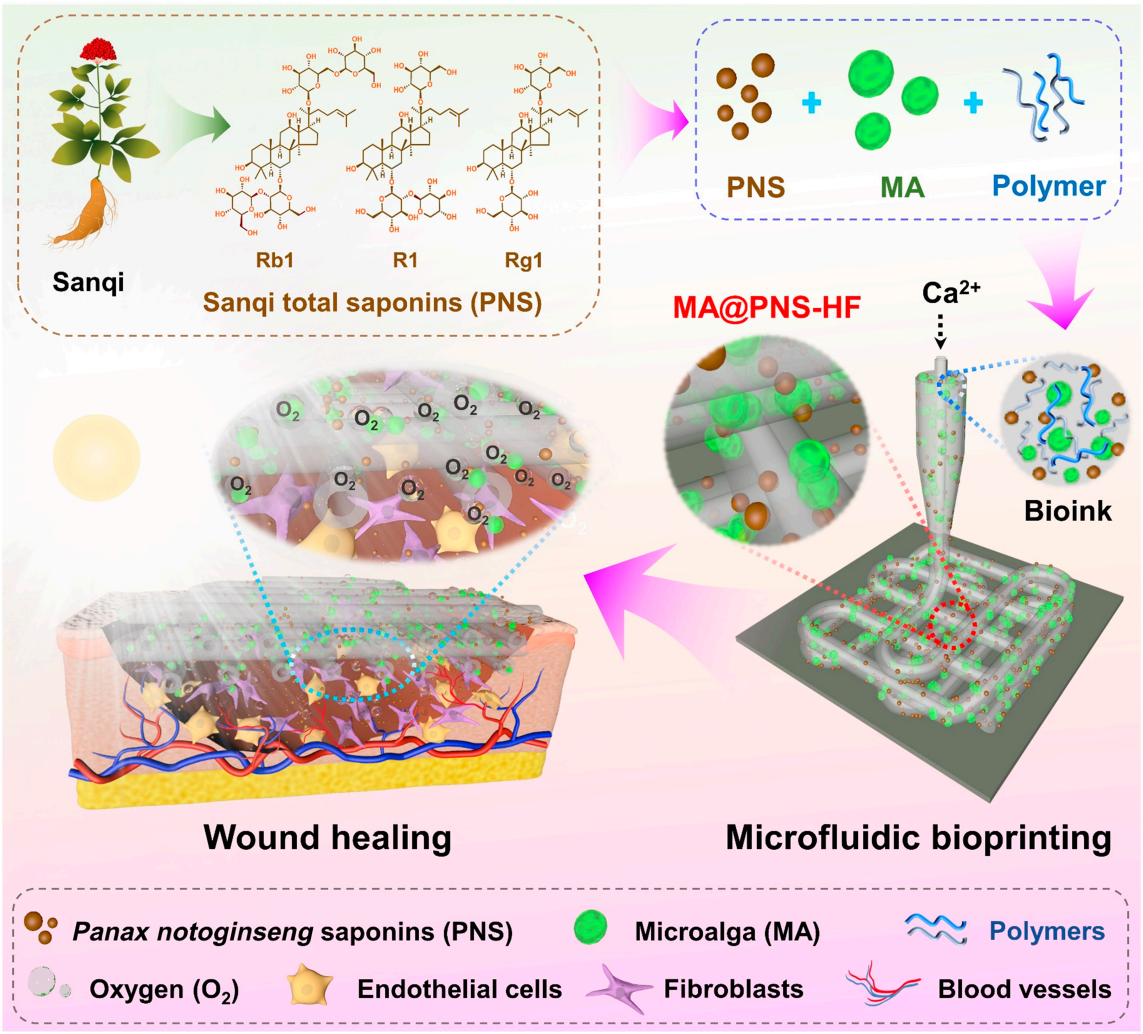
Schematic illustration of living Chinese herbal scaffolds from microfluidic bioprinting for wound healing. A traditional Chinese herb medicine (*Panax notoginseng* saponins [PNS]) and a living autotrophic microorganism (microalgae *Chlorella pyrenoidosa* [MA]) can be encapsulated into the MA@PNS-HF scaffolds via a facile microfluidic bioprinting strategy, and the obtained scaffolds could provide both PNS release and sustainable O_2_ supply for promoting wound healing.

Here, we present an innovative living Chinese herbal scaffold with the desirable features for wound healing via a microfluidic bioprinting method, as illustrated in Fig. 1. Traditional Chinese herbal medicines of great practical value for the treatment of wound healing and tissue repair have been recognized globally [27–29]. Among them, *Panax notoginseng* (known as Sanqi or Tianqi in Chinese) is one of the most frequently cultivated Chinese medicinal herbs, and *Panax notoginseng* saponins (PNS) are the primary effective compounds extracted from the herbal roots [30]. PNS contain multiple saponin constituents such as notoginsenoside R1, ginsenoside Rb1, Rg1, Rd, and Re [31], which are suggested by modern medical researches for inhibiting inflammation, promoting angiogenesis, and facilitating diabetic wound healing [32,33]. In contrast, as a new type of natural biological materials, algae have been recently employed in wound healing applications due to their unique nutritional value and highly efficient photosynthesis [34–37]. When the algae-based dressings were transplanted to wound sites, the living algae could continuously supply adequate oxygen for promoting wound healing through photosynthesis under light illumination [37]. Besides, the algae also contain some bioactive factors, which are favorable for cell proliferation and migration to further improve healing capacities [36]. Therefore, it is conceived that the incorporation of alive algae and PNS into biological scaffolds would provide sustainable oxygen supply and PNS release for the long-term wound healing process.

To this end, we adopted a microfluidic bioprinting method to fabricate the desired PNS-laden scaffolds (MA@PNS-HF) with alive microalgae (MA) encapsulated for accelerating diabetic wound healing (Fig. 1). An autotrophic unicellular microalga, namely, *Chlorella pyrenoidosa*, was chosen for the encapsulation due to its high photosynthetic efficiency and easy acquisition. The MA@PNS-HF scaffolds were generated by the extrusion-based coaxial microfluidic printing of the bioinks composed of MA, PNS, gelatin-methacryloyl (GelMA), and alginate biopolymers. The morphology and microstructure of the Chinese herbal scaffolds were characterized. Subsequently, the in vitro regenerative capacity of the obtained Chinese herbal scaffolds was explored. For photosynthetic oxygenation, alive MA cells were encapsulated into the Chinese herbal scaffolds and their protective effect against hypoxia was demonstrated in vitro. Furthermore, the in vivo regenerative capacity of the living Chinese herbal scaffolds was evaluated using a chronic wound model in diabetic mice.

## Results

### Fabrication and characterization of PNS-HF scaffolds

To start with, the PNS-HF scaffolds were prepared using an extrusion-based microfluidic printing method. As shown in Fig. S1, we firstly made a coaxial capillary microfluidic chip with an inner spindle injection capillary coaxially inserting into an outer tapered capillary, whose orifice diameters were 150 μm and 480 μm, respectively. An inner poly(vinyl alcohol) (PVA) solution containing low-concentration CaCl_2_ and an outer aqueous mixture containing PNS, GelMA, and alginate were pumped into the microfluidic chip. Owing to the fast ion-crosslinking between Ca^2+^ and alginate biopolymers, a continuous microfiber with a hollow channel was extruded from the chip, and further solidified by photo-crosslinking of GelMA biopolymers under ultraviolet (UV) irradiation. Specifically, the microfiber production was stable with a flat surface when the inner/outer flow rate was over 0.6, while a knot microfiber was formed when the ratio was less than 0.6, possibly ascribed to the insufficient Ca^2+^ diffusion from the inner solution (Fig. S2a). In addition, the hollow channels of the microfibers could be enlarged by decreasing the outer flow rates or increasing the inner flow rates (Fig. S2B and C). When the flow ratio was fixed at 1.0, a uniform microfiber with an outer diameter of ~315 μm could be obtained, whose inner hollow channel was about ~270 μm in diameter (Figs. S3 and S4).

**Fig. 2.**
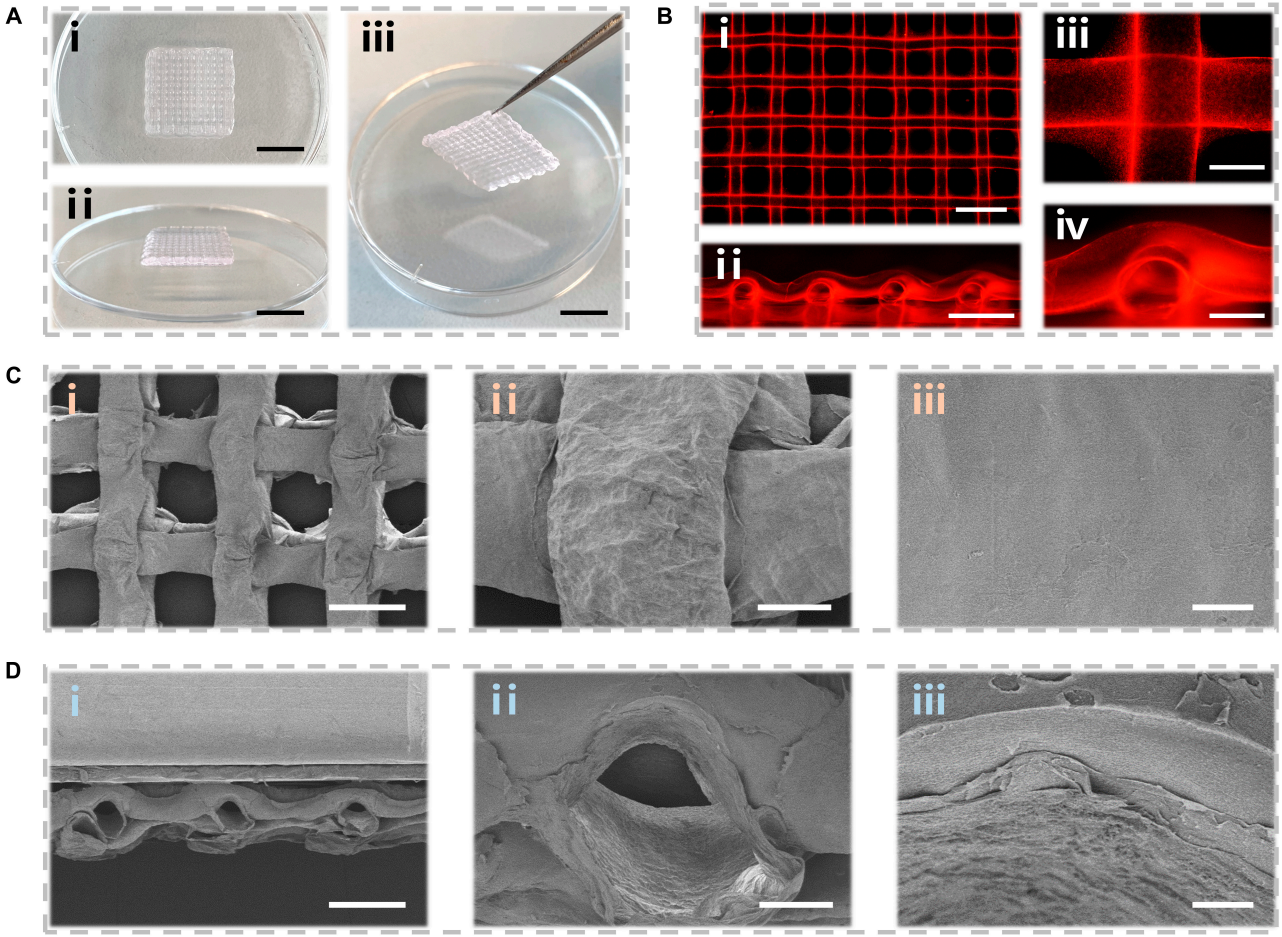
Characterization of the Chinese herbal scaffolds. (A) Photographs of the different views of PNS-HF scaffolds. Scale bars indicate 1 cm. (B) Fluorescent images of the PNS-HF scaffolds with hollow channels. Scale bars indicate 2 mm in (i), 1 mm in (ii), and 300 μm in (iii and iv). (C) Top and (D) sectional views of the SEM images of the freeze-dried PNS-HF scaffolds at different magnifications. Scale bars indicate 500 μm in (i), 100 μm in (ii), and 10 μm in (iii).

Next, we replaced the original printing nozzle with our homemade microfluidic chip in an extrusion-based 3D printer. Once the moving speed of the printer head well matched the extrusion rate of the hollow microfibers from the chip, a 3D scaffold could be printed on a flat plate through a typical layer-by-layer stacking process. The as-printed constructs could maintain their shapes in the air, and be easy to transfer using tweezers (Fig. 2A). Connection joints were formed between 2 layer-stacked hollow microfibers (Fig. 2B), probably because the solidification of the microfibers was incomplete before assembly into a 3D construct. Scanning electron microscopy (SEM) was performed to observe the surface and cross-section morphologies of lyophilized scaffolds. Both PNS-HF and non-loading hollow fibrous (HF) scaffolds displayed a smooth surface and a hollow channeled structure (Fig. 2C and D and Fig. S5). Notably, the scaffold displayed a highly ordered grid-like architecture with an interconnected porous structure (~600 μm, Fig. 2B) after printing, while the pore size decreased to about 400 μm after lyophilization (Fig.2C). In addition, Fourier transform infrared (FTIR) spectra of PNS-HF scaffolds displayed the characteristic bands of both the HF scaffolds and PNS powders, confirming the successful PNS incorporation into the PNS-HF scaffolds (Fig. S6). Furthermore, the PNS release behavior of PNS-HF scaffolds was investigated in comparison to that of the PNS-laden hydrogel bulks or hollow microfibers (Fig. S7). It was found that PNS could be gradually released from the PNS-laden hydrogel bulks and scaffolds, which was greatly different from the burst release from the PNS-laden microfibers, indicating that the microfluidic printing strategy enables the PNS release for a longer time from the as-printed fibrous scaffolds than the microfluidic spinning fibers. Taken together, the above results demonstrated that the Chinese herbal scaffolds were successfully fabricated from the microfluidic printing strategy. The HF scaffolds with interconnected porous structures could facilitate the exchange of gas and nutrient, and the diffusion and release of drug molecules [38,39], thus promoting cell survival and proliferation, migration, and tissue ingrowth into the scaffolds.

### In vitro regenerative properties of the PNS-HF scaffolds

**Fig. 3.**
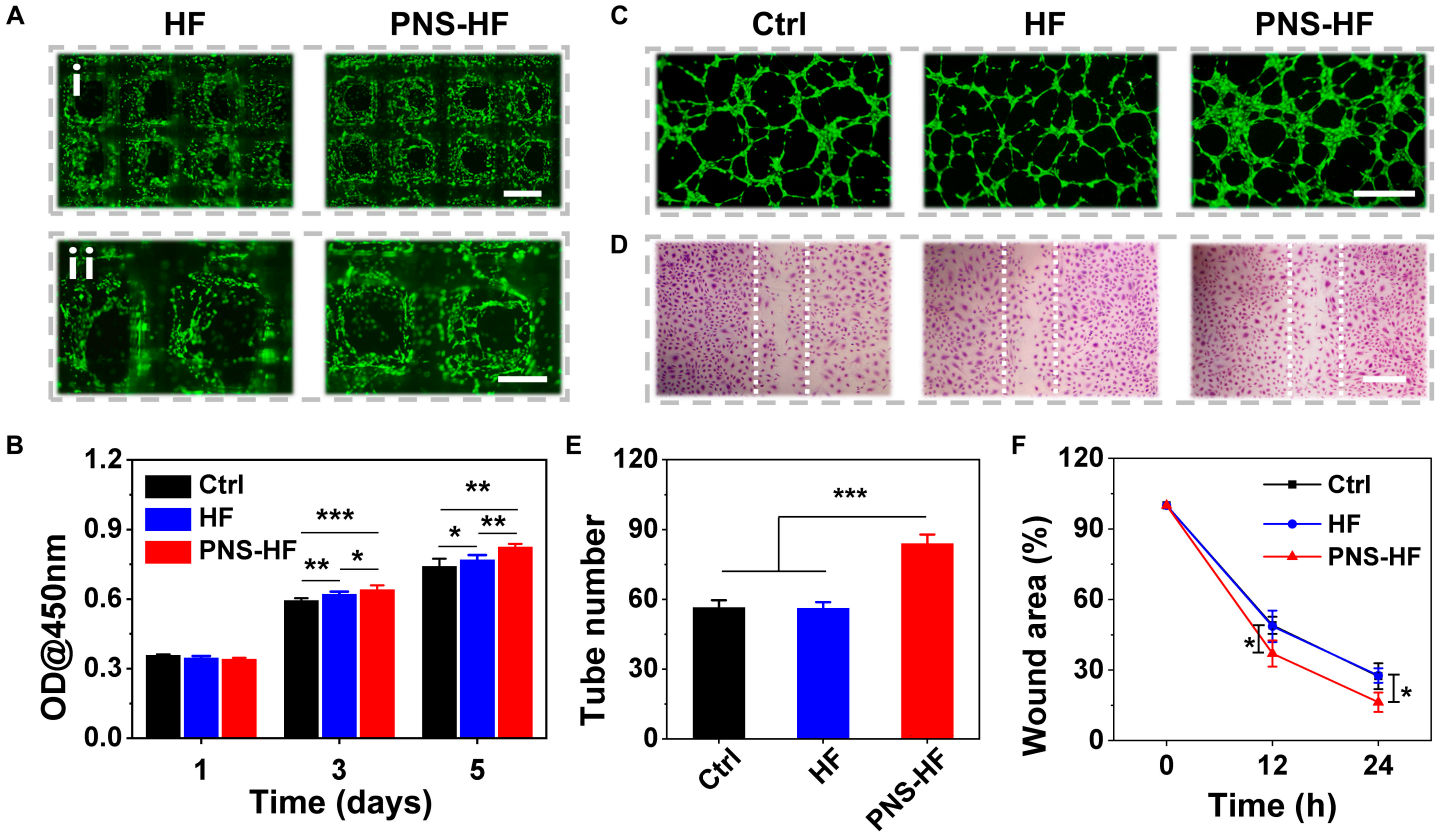
In vitro regenerative properties of the PNS-HF scaffolds. (A) Live/dead staining of HUVECs attached on the surface of HF and PNS-HF (PNS content: 25 μg/ml) scaffolds for 24 h. Alive or dead cells were stained in green or red, respectively. Scale bars indicate 1 mm in (i) and 500 μm in (ii). (B) CCK8 assay of HUVECs cultured with different scaffolds for 1, 3, and 5 days. Representative images of (C) the Matrigel tube formation for 6 h and (D) in vitro scratch assay of HUVECs cultured with different scaffolds for 24 h. White dotted lines indicate initial scratch edges at 0 h. Scale bars indicate 500 μm. Quantification of (E) the formed tubes and (F) the relative wound area of HUVECs in different groups. *N* = 6 per group, *P < 0.05, **P < 0.01, and ***P < 0.001.

Previous studies have demonstrated that free PNS with appropriate concentrations are capable of promoting cell proliferation, migration, and tubular structure formation in vitro [32,33]. Subsequently, we investigated the in vitro stimulatory effects of the PNS-HF scaffolds on human umbilical vein endothelial cells (HUVECs). For cell adhesion observation, the HF and PNS-HF scaffolds with HUVEC seeding were incubated for 24 h. Live/dead staining showed that the cells were well spread on the scaffold surface and almost all of them were alive as revealed with a strong green fluorescence (Fig. 3A). Owing to the gradual PNS release from the scaffolds, the HUVEC proliferation was obviously promoted in the PNS-HF group in comparison to the control and HF groups (Fig. 3B and Fig. S8). For proangiogenic capability evaluation, we performed a Matrigel tube formation assay by culturing the HUVECs with different scaffolds for 6 h. It was found that more vessel-like tubes were formed by HUVECs treated with PNS-HF scaffolds than those treated with HF scaffolds or without scaffold treatments (Fig. 3C and E). Furthermore, we conducted a typical scratch assay to explore the effect of PNS-HF scaffolds on cell migration. Our results showed that the PNS-HF scaffolds could significantly accelerate the scratch wound closure as compared to other groups (Fig. 3D and F). Altogether, these results indicated that the PNS could be gradually released from the PNS-HF scaffolds, exerting stimulatory effects on the cell proliferation, tube formation, and cell migration in vitro.

### Photosynthetic oxygen-generating capability of MA@PNS-HF scaffolds

**Fig. 4.**
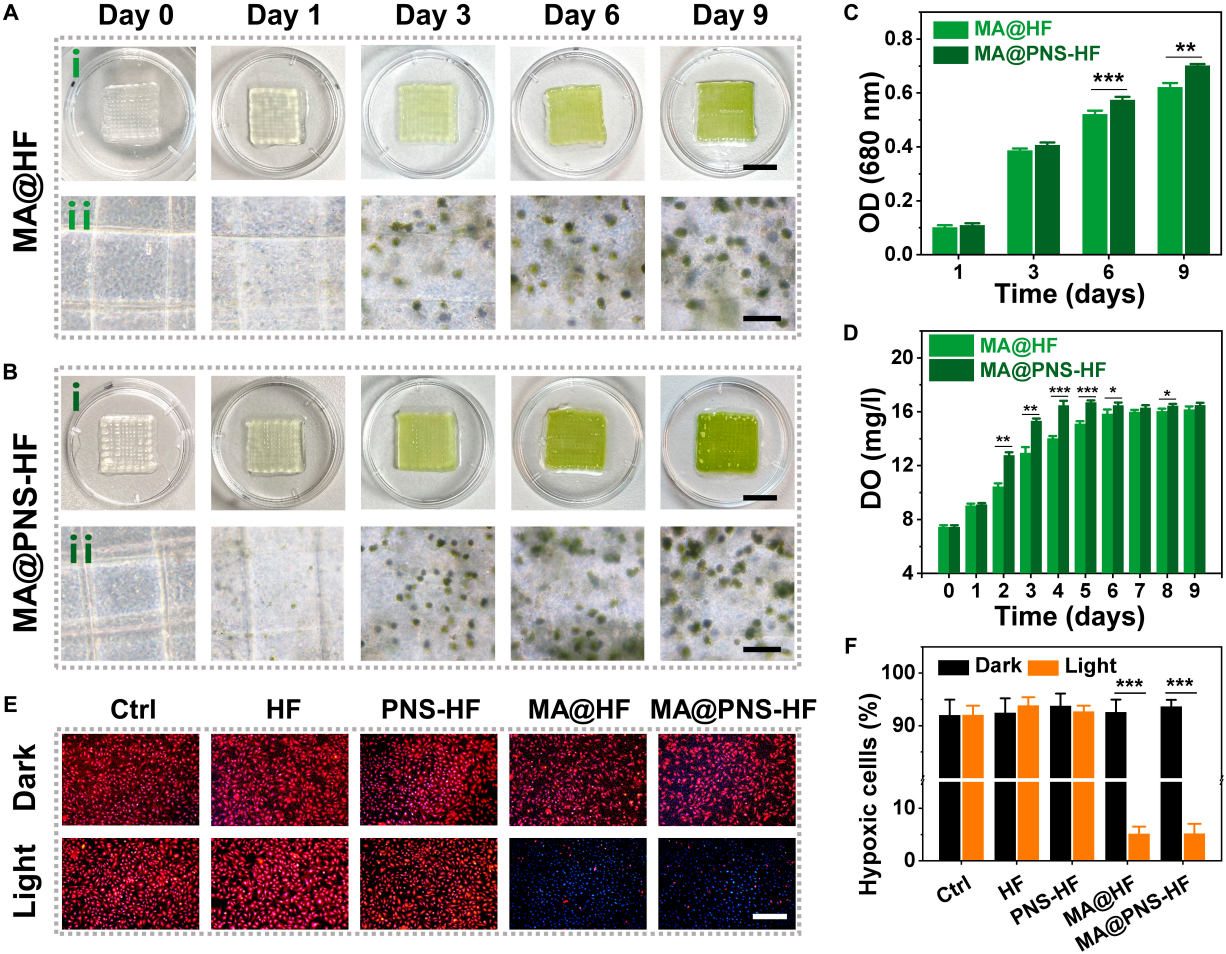
Photosynthetic oxygen-generating capability of living Chinese herbal scaffolds. (A and B) Digital photographs and corresponding bright-field microscopic images of MA-encapsulated HF (MA@HF, A) and MA-encapsulated PNS-HF (MA@PNS-HF, B) scaffolds cultivated at 25 °C for 9 days. Scale bars indicate 1 cm in (i) and 100 μm in (ii). (C) Chlorophyll contents of the microalgae within the scaffolds as represented by measuring OD (680 nm). (D) Dissolved oxygen (DO) release from the MA@HF and MA@PNS-HF scaffolds. (E) Fluorescent images of hypoxic HUVECs incubated with different living scaffolds for 3 days under dark or light conditions, and double stained with the hypoxia probe ([Ru(dpp)_3_]Cl_2_, red) and DAPI (blue). (F) Percentages of hypoxic HUVECs cultured in different conditions for 3 days. *N* = 6 per group, *P < 0.05, **P < 0.01, and ***P < 0.001.

Having confirmed the biocompatibility of the PNS-HF scaffold, we decided to incorporate the living MA into the herbal scaffold for additional photosynthetic oxygenation capacity. After bioprinting, the MA@HF and MA@PNS-HF scaffolds were cultured in light at 25 °C for 9 days. While the MA-loading constructs were almost colorless on day 0, they gradually appeared in green after 9 days of cultivation, and the growth of MA within bioprinted scaffolds was observed microscopically (Fig. 4A and B). The MA viability was further confirmed by measuring the chlorophyll contents and dissolved oxygen release from the MA@HF and MA@PNS-HF scaffolds, both of which revealed a gradual increase of the MA density over the culture period of 9 days (Fig. 4C and D). Notably, the chlorophyll content of MA@PNS-HF scaffolds was significantly higher than that of MA@HF scaffolds, indicating that PNS could play a positive role in MA growth.

Oxygen plays an important role in maintaining normal cell respiration, promoting cell proliferation and migration, regulating angiogenesis, and interacting with a variety of cytokines [40]. In addition, oxygen is also necessary for the synthesis of hydroxyproline, which is a unique component in collagen fibers during wound remodeling [41,42]. It is estimated that tissue oxygen tension of at least 20 mmHg is required for a wound to heal, while the oxygen tension is measured as low as 5 mmHg in a non-healing wound [40]. Therefore, adequate oxygen supply is highly desirable for the treatment of chronic hypoxia wounds. Given that MA can actively undergo photosynthesis to release oxygen, we further investigated the ability of the living photosynthetic scaffolds to modulate cellular hypoxic conditions. The MA@HF or MA@PNS-HF scaffolds were co-cultured with HUVECs for 3 days in 1% oxygen conditions. The intracellular hypoxic conditions can be visualized using [Ru(dpp)_3_]Cl_2_, which is a commonly used hypoxic indicator with a red fluorescence that can be quenched by oxygen [35]. Our result showed that the red fluorescence in HUVECs after treatment with MA@HF or MA@PNS-HF scaffolds under light conditions was remarkably reduced, greatly different from the majority of hypoxic cells observed in the other control groups (Fig. 4E and F), which could be attributed to the photosynthetic oxygen-generating capability of the MA in the scaffolds. Similarly, the cell viability of hypoxic HUVECs incubated with MA@HF and MA@PNS-HF scaffolds under light conditions was significantly higher than the cells kept under dark conditions (Fig. S9), further confirming that the living photosynthetic scaffolds could effectively protect cells against hypoxia damage.

The degradation of the scaffold over time also plays a crucial role in wound healing and drug release. Besides, the frequent change or removal of undegradable wound dressings may cause secondary injury to the wounds. Thus, the in vitro degradation behavior of the living Chinese herbal scaffolds was investigated by immersing the HF, PNS-HF, MA@HF, and MA@PNS-HF scaffolds in phosphate-buffered saline (PBS) at 37 °C for 14 days. The scaffolds were photographed and weighed as shown in Fig. S10. During the PBS immersion process, it was found that all scaffolds gradually lost their integrity with residual weight fractions less than 15% on day 14. These results indicate that the living Chinese herbal scaffolds are degradable in a physiological microenvironment, which is favorable for further in vivo wound healing applications.

### In vivo wound healing ability of MA@PNS-HF scaffolds

**Fig. 5.**
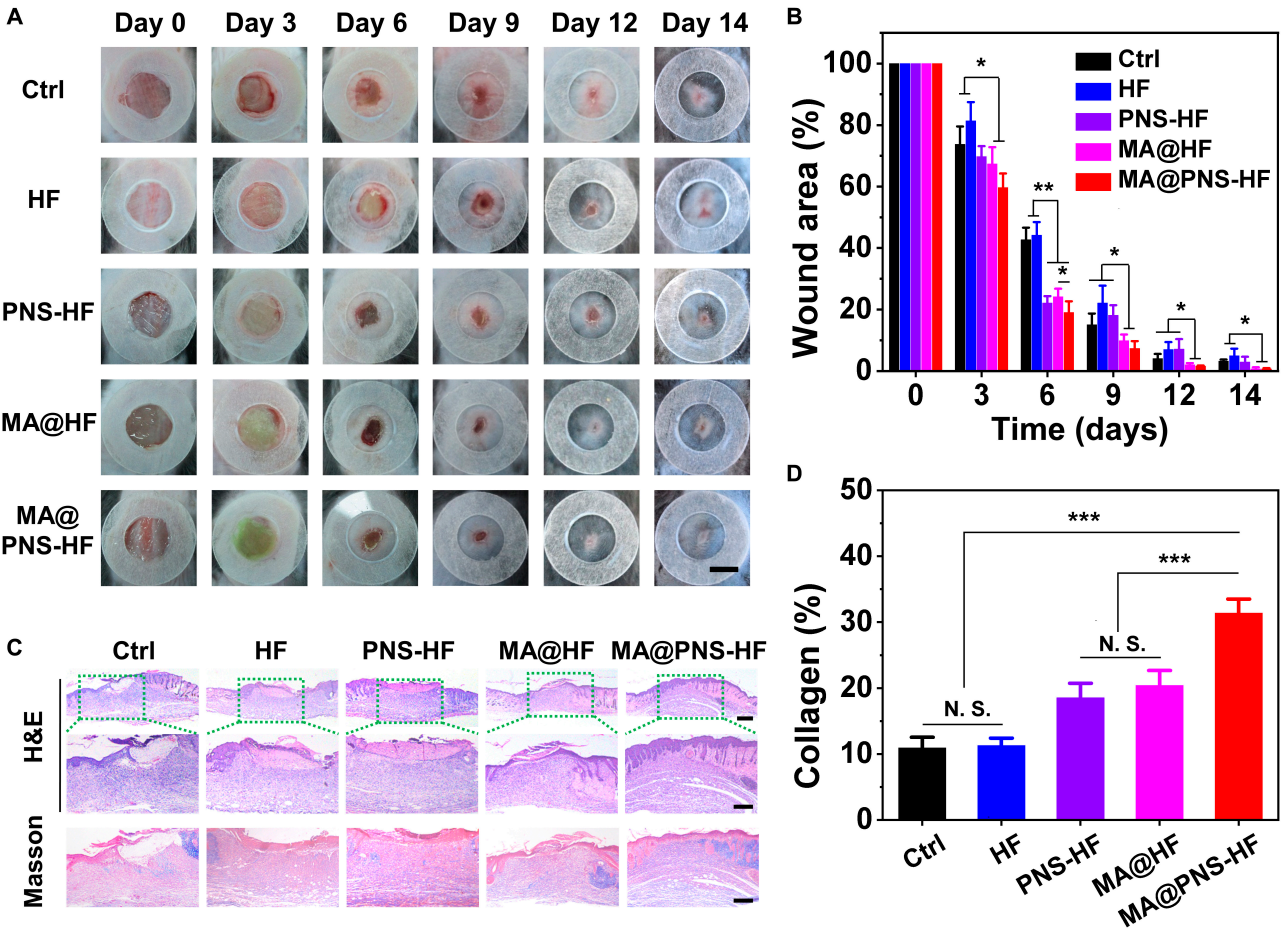
Accelerated chronic wound healing by living Chinese herbal scaffolds. (A) Representative photographs of the wound closure on days 0, 3, 6, 9, 12, and 14 after different treatments. The scale bar indicates 5 mm. (B) Semi-quantification of relative wound area in different groups for 14 days. (C) Representative H&E and Masson staining images of different groups on day 14. Scale bars indicate 500 μm (top) and 300 μm (middle, bottom). (D) Semi-quantification of the collagen deposition in different groups. *N* = 6 per group, N.S. indicates no significance, and *P < 0.05, **P < 0.01, and ***P < 0.001.

To investigate the in vivo wound healing ability of the living MA@PNS-HF scaffolds, full-thickness skin wounds were created in the streptozotocin-induced diabetic mice. An HF, PNS-HF, MA@HF, or MA@PNS-HF scaffold was then implanted into the wounds. The wound healing process was recorded for 14 days. It should be noted that the scaffold could well adhere to the wounds during the in vivo experimental period. For the first 6 days, we illuminated the living algae-containing scaffolds to generate oxygen at the wound beds via photosynthesis. As shown in Fig. 5A, the MA@HF and MA@PNS-HF scaffolds gradually turned green, indicating that the encapsulated algae maintained their proliferation ability at wound beds. From day 6, a dark and dry scaffold–scab mixture was observed at the wound sites, which may not be an appropriate condition for the MA living. Thus, we stopped light illumination from day 6. In addition to the oxygen release from the scaffolds, the scaffold–scab mixture could protect wounds and release herbal drugs from the gradually degraded scaffolds at the wound beds. Therefore, the wounds healed faster in PNS-HF, MA@HF, and MA@PNS-HF groups than those in HF and Ctrl groups. After 14 days, almost all skin wounds reached complete closure in the MA@HF and MA@PNS-HF group, in remarkable contrast to the visible wounds in other groups.

Semi-quantitative analysis showed that the relative wound area on day 14 in the Ctrl, HF, PNS-HF, MA@HF, and MA@PNS-HF groups was 3.2 ± 0.5%, 5.0 ± 2.3%, 2.9 ± 1.7%, 0.7 ± 0.5%, and 0.7 ± 0.3%, respectively (Fig. 5B). It was found that there were no significant differences between the 2 MA-laden scaffold groups, indicating the remarkable efficacy of living MA to improve wound closure rate probably due to the photosynthetic oxygenation at the early stages of healing. Hematoxylin–eosin (H&E) and Masson’s Trichrome staining was performed on day 14 (Fig. 5C). Incomplete epidermis and large residual scabs could still be observed in the Ctrl, HF, and PNS-HF groups, indicating delayed wound healing. By contrast, clear stratified epidermis layers and better collagen deposition were found in the 2 living MA-laden scaffold groups. Semi-quantitative analysis of the collagen deposition showed that the MA@PNS-HF group had the highest extents of collagen deposition (31.3 ± 2.1%), compared with those in the control (10.9 ± 1.6%), HF (11.3 ± 1.1%), PNS-HF (18.5 ± 2.2%), and MA-HF (20.4 ± 2.3%) groups (Fig. 5D). The best chronic diabetic wound healing efficiency of MA@PNS-HF scaffolds could be attributed to the synergistic effects of oxygenating MA in the early stage and gradual PNS release over the entire healing process.

**Fig. 6.**
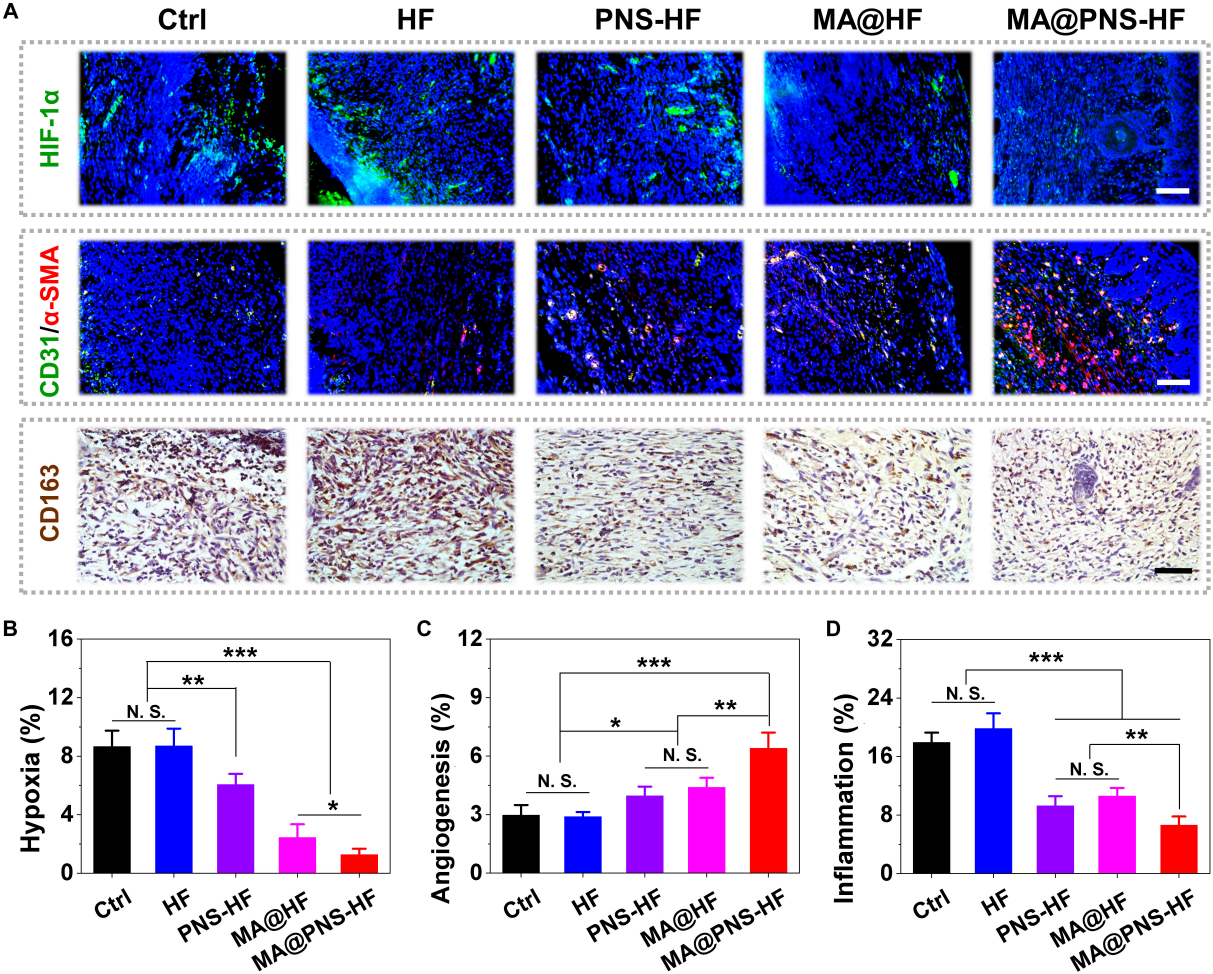
Alleviated hypoxia, promoted angiogenesis, and reduced inflammation by living Chinese herbal scaffolds in vivo. (A) Representative histological images of HIF-1α, CD31&α-SMA, and CD163 staining of skin tissue sections from different groups on day 14. Scale bars indicate 200 μm (top, middle) and 50 μm (bottom). Semi-quantification of (B) HIF-1α, (C) CD31, and (D) CD163 staining on day 14. *N* = 6 per group, N.S. indicates no significance, and *P < 0.05, **P < 0.01, and ***P < 0.001.

Wound hypoxia and impaired vascularization are known as the major causes of non-healing of wounds in patients with diabetes [1,4,43]. To investigate whether the oxygenating scaffolds could affect the local hypoxia, angiogenesis, and inflammation states in diabetic wounds, we conducted immunohistochemical analysis of hypoxia-inducible factor 1-alpha (HIF-1α), cluster of differentiation 31 (CD31), and CD163 staining (Fig. 6A). The tissue hypoxia was revealed by HIF-1α immunofluorescence. Benefiting from the photosynthetic oxygenation capacity of living MA within the scaffolds, the local hypoxia could be effectively relieved by the MA@HF and MA@PNS-HF scaffolds. As shown in Fig. 6B, the expression levels of HIF-1α were significantly lower in the MA@HF (2.5 ± 0.9%) and MA@PNS-HF (1.3 ± 0.4%) groups than those in the Ctrl (8.7 ± 1.1%), HF (8.7± 1.2%), and PNS-HF (6.1 ± 0.7%) groups. Additionally, the angiogenesis was examined using CD31& α-smooth muscle actin (α-SMA) double immunofluorescence staining, and the average microvessel densities were quantified using CD31 immunostaining (Fig. 6C). As a result, the vessel densities of the PNS-HF (4.0 ± 0.5%) and MA@HF (4.4 ± 0.5%) groups were significantly higher than those of the Ctrl (3.0 ± 0.5%) and HF (2.9 ± 0.2%) groups, further confirming proangiogenic effects of either oxygenating MA or PNS stimulation for accelerated wound healing as previously reported [32,33,37]. Notably, microvessel formation in the MA@PNS-HF group (6.4 ± 0.8%) was the highest among all groups, which could be attributed to the synergistic effects of both sustainable oxygen supply and PNS release from the MA@PNS-HF scaffolds. Based on the in vivo functional and histological evaluation, we could cautiously conclude that the incorporation of the oxygen-generating MA and the Chinese herb PNS in the hydrogel scaffolds could effectively alleviate tissue hypoxia and promote vascularization in diabetic wounds.

In addition, Fig. 6D shows the percentage of cells that expressed CD163 (a specific marker of activated macrophages [6]) to evaluate the inflammatory response of the scaffolds in mice. A lowest number of CD163 positive cells (6.6 ± 1.2%) was found in the MA@PNS-HF group, and both the PNS-HF (9.3 ± 1.3%) and MA@HF (10.6 ± 1.1%) groups had a significantly lower proportion of cells that expressed CD163 markers than that in the control (17.9 ± 1.3%) and HF (19.8 ± 2.1%) groups. These data revealed that the implantation of MA-laden and/or PNS-containing scaffolds could relieve the local inflammatory reaction in diabetic wounds. Taken together, the above findings suggested that the living MA@PNS-HF scaffolds could effectively accelerate diabetic wound healing by reducing inflammation, alleviating tissue hypoxia, and promoting vascularization.

## Discussion

Wound healing, especially the healing of chronic wounds and extensive injures, has been a major public concern to social and healthcare systems worldwide [1,2]. Traditional skin grafts including autografts, allografts, and xenografts usually suffer from intrinsic and extrinsic limitations regarding availability, immunologic rejection, and risks of morbidity [20,44]. Alternatively, bioengineered skin substitutes or advanced dressing materials have been developed and demonstrated to be useful in wound healing via the combination of biological scaffolds, growth factors, and mammalian cells such as keratinocytes, fibroblasts, endothelial cells, stem cells, platelets, or platelet bioproducts [10,20]. However, one common challenge in these approaches is the insufficient supply of nutrients and oxygen to the host cells, which is the prerequisite for the survival and viability of the skin substitute after transplantation [36]. By contrast, our current study offers a simple and cost-effective alternative strategy for sustainable oxygen and nutrient supply for promoting chronic wound healing. We believe that the obtained living Chinese herbal scaffolds can set a stage for future research on the development of engineered living materials for skin regeneration. For example, symbiotic cell constructs can be developed via incorporating both oxygen-generating microorganisms and mammalian cells to prolong the survival of the host cells under hypoxic conditions, and thereby promote the wound healing rate and quality. For the treatment of deep wound healing, a critical issue would be the insufficient light penetration for photosynthesis [45,46]. Hence, further work should overcome this issue, and near-infrared light with a deep-tissue-penetrating ability might be employed to power the photosynthetic microorganisms [47,48]. In addition, the customized microfluidic 3D bioprinting platform can further be adapted for the fabrication of specific patient-needing wound dressings with desired geometries, compositions, and functionalities for various wound types.

In summary, we have presented the microfluidic bioprinting of living Chinese herbal scaffolds with drug delivering ability and photosynthetic oxygen-generating capability for promoting chronic wound healing. Owing to the herbal drug release and autotrophic photosynthetic oxygenation, the obtained scaffolds could significantly improve cellular activity in vitro and accelerate wound closure in diabetic mice. Therefore, our present work demonstrates the great potential of living Chinese herb scaffolds for diabetic wound healing, and the strategy proposed here may also be practical in other impaired tissue repair applications.

## Materials and Methods

### Materials

Sodium alginate (SA) and calcium chloride (CaCl_2_) were obtained from Alfa Aesar. Lithium phenyl-2,4,6-trimethylbenzoylphosphinate (LAP), GelMA, and PVA were purchased from Shanghai Aladdin Bio-Chem Technology. PNS (i.e., Sanqi total saponins), and the LIVE/DEAD Cell Imaging kit were purchased from Solarbio Life Sciences. *C. pyrenoidosa* and their culture medium were bought from Nanjing Health Biotech. HUVECs and endothelial cell medium (ECM) were received from ScienCell. Cell counting kit-8 (CCK8) was bought from Beyotime Biotechnology. Growth Factor Reduced Matrigel was purchased from Corning Life Sciences. Deionized water (18.2 MΩ·cm^−1^) was acquired from a Milli-Q water purification system.

### Microfluidic spinning of PNS-laden hollow microfibers

To generate the PNS-laden hollow microfibers (PNS-HF), a capillary microfluidic chip was custom-made by assembling 2 cylindrical glass capillaries on a piece of glass slide. As shown in Fig. S1, a spindle-shaped capillary was coaxially inserted into a tapered capillary, and their orifice diameters were 150 μm and 480 μm, respectively. The outer phase was aqueous pre-gel solution containing PNS (25 μg/ml), GelMA (5% w/v), SA (2.5% w/v), and LAP (0.1% w/v), while the inner phase was PVA solution (10% w/v) containing CaCl_2_ (0.8% w/v). Both the outer and inner input flow rates were 3 ml/h (inner/outer flow ratio = 1.0) during the microfluidic spinning process. All fluids were pumped into the microfluidic chip and the obtained hollow microfibers were collected in a CaCl_2_ bath (2% w/v). The hollow microfibers were primarily formed by the fast ion-crosslinking between Ca^2+^ and SA. Afterwards, the photocrosslinking of GelMA was induced under UV irradiation (365 nm, 100 W, 5 min). The microfibers were kept in 2% CaCl_2_ overnight before further use.

### Microfluidic bioprinting of living microalgae-encapsulated PNS-HF scaffolds

The homemade coaxial capillary microfluidic chip was employed to substitute the original printing head in a programmable extrusion-based 3D printer. The living microalgae-encapsulated MA@PNS-HF scaffolds were directly printed in a dry flat plate during the microfluidic 3D printing process. The overall shape, scaffold size, and micropore structure were pre-determined by the 3D models. An exact match was required between the extrusion rate of microfluidic fibers and the nozzle moving speed of the programmable printer, ensuring the successful microfluidic printing of MA@PNS-HF scaffolds. The extrusion rate of microfluidic fibers could be effectively controlled by the syringe pumps. After a layer-by-layer printing process, 3D-stacked constructs with straight hollow struts and interconnected macropores between the struts could be obtained.

For microfluidic bioprinting of a typical MA@PNS-HF scaffold, the inner fluid was PVA solution (10% w/v) containing CaCl_2_ (0.8% w/v), while the outer fluid was aqueous pre-gel solution containing MA (10^6^ cells/ml), PNS (25 μg/ml), GelMA (5% w/v), SA (2.5% w/v), and LAP (0.1% w/v). The input flow rates of both the inner and outer fluids were 3 ml/h. The printing time and nozzle moving speed were 3 min and 6 mm/s, respectively. The scaffold size was pre-determined by the 3D model as 2 cm × 2 cm × 2 mm, while the distance between 2 adjacent fibers was set to 1 cm, and the height between layers was set to 400 μm. The hollow channel of MA@PNS-HF scaffold struts was initially formed during printing due to the ion-crosslinking between SA in the outer phase and Ca^2+^ ions diffused from the inner phase. Strengthened gelation of MA@PNS-HF scaffolds was obtained after printing by GelMA photocrosslinking under UV irradiation for 5 min and SA crosslinking in the CaCl_2_ bath (2% w/v) for 30 min. All chemicals, fluids, and microfluidic devices were either filtrated through a 0.22-mm sterile filter or exposed to UV irradiation for 12 h before biological experiments. After bioprinting, the scaffolds were further immersed in 75% ethanol for 30 min and rinsed 3 times with PBS for cell culturing or in vivo wound dressing.

### Characterizations

The hollow microfibers and scaffolds were examined under a stereomicroscope (Olympus BX51, Tokyo, Japan), and optical bright-field and fluorescent images were obtained. The hydrogel samples were further lyophilized and their microstructures were observed by a field emission scanning electron microanalyzer (FE-SEM, SU8010, Hitachi, Japan). The infrared spectra of the PNS powder, lyophilized HF, and PNS-HF scaffolds were acquired using an attenuated total reflectance Fourier transform infrared spectroscope (ATR-FTIR, Tensor II, Bruker, Germany).

### In vitro PNS release from the PNS-HF scaffolds

For in vitro drug release assay, a PNS-HF scaffold (PNS content: 25 μg/ml, scaffold size: 20 mm × 20 mm × 2 mm) was immersed in PBS solution (2 ml) at 37 °C. Meanwhile, PNS-laden hollow microfibers and hydrogel bulks with equal volumes were also treated for comparison. One milliliter of PBS solution was aspirated and centrifuged at specific time points. The PNS concentration was determined by measuring the absorbance with a UV–Vis spectrophotometer at 205 nm. One milliliter of fresh PBS solution was supplemented afterwards, and the drug release assay was conducted for 120 h. The cumulative drug release percentage was calculated as follows: Drug release (%) = Cumulative released drug amount/Total drug amount × 100%.

### Cell adhesion and proliferation assay

Typically, HUVECs were cultured in a humidified incubator (5% CO_2_, 37 °C). For cell adhesion assay, HUVECs (1 × 10^5^ per well) were seeded on the 24-well plates containing HF or PNS-HF scaffolds (scaffold size: 20 mm × 20 mm × 2 mm, PNS content: 25 μg/ml). The cells and scaffolds were stained using a LIVE/DEAD Cell Imaging kit after 24 h of culture. For proliferation assay, the HUVECs were incubated with HF or PNS-HF scaffolds for 5 days, and CCK8 assay was conducted every 2 days. For a typical CCK8 assay, the culture medium was replaced with fresh medium containing the CCK8 kit (10% v/v). The medium was pipetted after incubation of 2 h, and the absorbance at 450 nm was measured via a microplate reader (Epoch, BIO-TEK).

### Tube formation assay

HUVECs (5 × 10^4^ cells/well) were seeded on a 24-well plate with Matrigel matrix coating (50% v/v in ECM, 250 μl per well). The transwell inserts laden with HF or PNS-HF scaffolds (scaffold size: *Ф* 5 mm × 2 mm, PNS content: 25 μg/ml) were gently transferred into the plate. After 6 h of incubation, the transwell inserts were removed, and the cells were stained with Calcein-AM. A fluorescence microscope (Zeiss) was then used to observe the tube formation of HUVECs.

### Scratch wound healing assay

HUVECs (1 × 10^5^ cells/well) were seeded in a 24-well plate and incubated overnight. A single scratch was made on the cells with a sterile 200-μl pipette tip. The plate was rinsed with PBS twice to remove the unattached cells. Then, transwell inserts laden with HF or PNS-HF scaffolds (scaffold size: *Ф* 5 mm × 2 mm, PNS content: 25 μg/ml) were transferred into the plate. At appropriate time points, the cells were photographed, and ImageJ software was used to measure the wound areas. The wound closure was quantified as follows: Relative wound area (%) = W_t_/W_0_ × 100%. Here, Wt represents the wound area at specific time points, while W_0_ represents the wound area immediately after scratching.

### Photosynthetic oxygen release from living Chinese herbal scaffolds

For the evaluation of the photosynthetic oxygenating capacity, MA@HF and MA@PNS-HF scaffolds (PNS content: 25 μg/ml, MA concentration: 10^6^ cells/ml, scaffold size: 20 mm × 20 mm × 2 mm) were illuminated by a light-emitting diode (LED) light bulb (light intensity: 6,000 lux, distance: 10 cm). The microalgae growth within the scaffolds at 25 °C was recorded for 9 days. The photosynthetic oxygen release from the scaffolds was monitored in real time using an oxygen microsensor when the scaffolds were under continuous light illumination. Chlorophyll contents in the bioprinted scaffolds were determined using an ethanol extraction method. Typically, to extract green pigments, the whole scaffold was immersed in 95% ethanol under gentle shaking or 6 h and centrifuged at 5,000 revolutions per minute for 5 min at 25 °C, and the supernatants were collected. The absorbance at 680 nm was measured via a microplate reader.

### In vitro hypoxic alleviation by the Chinese herbal living scaffolds

The in vitro hypoxic alleviation efficiency of MA@HF and MA@PNS-HF scaffolds was explored using [Ru(dpp)_3_]Cl_2_ as a hypoxia indicator, whose red fluorescence might be quenched by the generated oxygen from the scaffolds. HUVECs (10^5^ cells/well) were pre-seeded in a 24-well plate and incubated overnight in a normal incubator. For hypoxic simulation, the cells were transferred to a humidified incubator containing 94% N_2_, 5% CO_2_, and 1% oxygen. A transwell insert laden with an HF, PNS-HF, MA@HF, or MA@PNS-HF scaffold (PNS content: 25 μg/ml; scaffold size: *Ф* 5 mm × 2 mm, MA concentration: 10^6^ cells/ml) was transferred into the cell culture plate, which was kept under hypoxic conditions for 72 h. For the light groups, the scaffolds were continuously exposed to an LED bulb (light intensity: 6,000 lux, distance: 10 cm). For the dark groups, the samples were kept in the dark. Seventy-two hours after different treatments, the scaffolds were removed from all plates. For cell morphology observation, one-third of the cell samples were stained with a LIVE/DEAD Cell Imaging kit. For cellular viability quantification, one-third of the samples were evaluated with CCK8 assay. For hypoxic detection, the remaining cells were pre-incubated with [Ru(dpp)_3_]Cl_2_ (8 μg/ml, red) and then subjected to all treatments. The cells were fixed in 4% paraformaldehyde and rinsed 3 times with PBS, and the cell nuclei were further stained with 4′,6-diamidino-2-phenylindole (DAPI, blue) for 5 min. All fluorescent samples were observed by a fluorescence microscope (Zeiss).

### In vitro degradability of the living Chinese herbal scaffolds

The initial weights of HF, PNS-HF, MA@HF, and MA@PNS-HF scaffolds (PNS content: 25 μg/ml, MA concentration: 10^7^ cells/ml; size: 20 mm × 20 mm × 2 mm) were weighed before being completely immersed in 3 ml of PBS in the dark at 37 °C for 14 days. The scaffolds were photographed and weighed after wiping out water on the scaffold surface on days 0, 3, 7, 10, and 14. The residual weight fraction was calculated as follows: residual weight fraction (%) = W_t_/W_0_ × 100%. Here, W_t_ represents the scaffold weights at certain time points, and W_0_ represents initial weights.

### In vivo chronic wound healing evaluation

C57BL/6 mice (male, 4 to 6 weeks, 22 to 25 g) were acquired from Beijing Vital River Laboratory Animal Technology Co., Ltd. The experimental protocol was approved by the Animal Care and Use Committee of Wenzhou Institute, University of Chinese Academy of Sciences (No. WIUCAS22021102, Zhejiang, China). A type I diabetic mouse model was established via intraperitoneal injection of streptozotocin according to previously reported methods [8,37]. Each mouse received an intraperitoneal injection of streptozotocin with a dosage of 50 mg/kg every 3 days for 5 times. The blood glucose level was monitored every 3 days for 4 weeks. After that, for in vivo diabetic wound healing assay, we selected the diabetic mice with blood glucose levels over 20 mM and created a full-thickness round wound (*Φ* 8 mm) on the shaved dorsal skin in each mice.

The wounded mice were randomly grouped (*n* = 8): (a) Ctrl group (without scaffold implantation), (b) HF group, (c) PNS-HF group, (d) MA@ HF group, and (e) MA@PNS-HF group. For the scaffold-implanted groups, an HF, PNS-HF, MA@ HF, or MA@PNS-HF scaffold (*Φ*8 mm × 2 mm) was implanted into the wounds, which were further covered by an transparent adhesive bandage (3M). We did not change the scaffolds during the in vivo experimental period. The changes of both the scaffolds and skin wounds were monitored and photographed for 14 days. For the first 6 days, we illuminated the living algae-containing scaffolds to generate oxygen at the wound beds via photosynthesis. From day 6, a dark and dry scaffold–scab mixture was observed at the wound sites, which may not be an appropriate condition for algae living; hence, we stopped light illumination from day 6.

The skin wounds were photographed and measured using ImageJ software. The wound healing efficiency was semi-quantified as follows: relative wound area (%) = W_t_/W_0_ × 100%. Here, W_t_ represents the wound area at specific time points, while W_0_ represents the wound area immediately after wounding. Almost all skin wounds reached complete closure in the MA@PNS-HF group on day 14, so we sacrificed the mice and harvested the skin tissues for histological analysis. Typical H&E and Masson’s Trichrome staining, and immunohistochemical analysis of HIF-1α, CD31, and CD163 staining were performed to investigate the microstructure of the regenerated skin tissues, tissue hypoxia, angiogenesis, and inflammation response in the wounds.

### Statistical analysis

Data are displayed as means ± standard deviations (*n* ≥ 4). All graphs were created from OriginPro 2020 software. Two-tailed unpaired Student’s *t* tests were performed to calculate the statistical significance between 2 groups, and a *P* value < 0.05 was considered significant (*P < 0.05, **P < 0.01, and ***P < 0.001).

## Data Availability

All data are available in the main text or the Supplementary Materials.

## Supplementary Materials

Supplementary 1 Fig. S1. Photograph and optical image of the custom-made coaxial capillary microfluidic chip. Fig. S2. Different hollow microfibers generated from microfluidic spinning at the varied inner-to-outer flow ratios from 0.2 to 2.0. Fig. S3. Top and sectional views of optical and fluorescent micrographs of the hollow microfibers. Fig. S4. Top and sectional views of SEM morphologies of the hollow microfibers at different magnifications. Fig. S5. Top and sectional views of SEM morphologies of the HF scaffolds without PNS loading at different magnifications. Fig. S6. FTIR spectra of the PNS powder, HF, and PNS-HF scaffolds. Fig. S7. PNS release profiles of the PNS-laden hydrogel bulks, hollow microfibers, and scaffolds in PBS at 37 °C. Fig. S8. Live/dead staining of HUVECs cultured with HF and PNS-HF scaffolds. Fig. S9. Live/dead staining and cell viability of HUVECs cultured with HF, PNS-HF, MA@HF, and MA@PNS-HF scaffolds for 3 days under 1% oxygen conditions. Fig. S10. In vitro degradation of HF, PNS-HF, MA@HF, and MA@PNS-HF scaffolds in PBS solution at 37 °C for 14 days.
