# Supplementary material for: Living Chinese Herbal Scaffolds from Microfluidic Bioprinting for Wound Healing
Source: Research (Wash D C). 2023 May 9;6:0138. doi: 10.34133/research.0138 (PMC10204746; doi:10.34133/research.0138)
Supplement: Supplementary 1 — Fig. S1. Photograph and optical image of the custom-made coaxial capillary microfluidic chip. Fig. S2. Different hollow microfibers generated from microfluidic spinning at the varied inner-to-outer flow ratios from 0.2 to 2.0. Fig. S3. Top and sectional views of optical and fluorescent micrographs of the hollow microfibers. Fig. S4. Top and sectional views of SEM morphologies of the hollow microfibers at different magnifications. Fig. S5. Top and sectional views of SEM morphologies of the HF scaffolds without PNS loading at different magnifications. Fig. S6. FTIR spectra of the PNS powder, HF, and PNS-HF scaffolds. Fig. S7. PNS release profiles of the PNS-laden hydrogel bulks, hollow microfibers, and scaffolds in PBS at 37 °C. Fig. S8. Live/dead staining of HUVECs cultured with HF and PNS-HF scaffolds. Fig. S9. Live/dead staining and cell viability of HUVECs cultured with HF, PNS-HF, MA@HF, and MA@PNS-HF scaffolds for 3 days under 1% oxygen conditions. Fig. S10. In vitro degradation of HF, PNS-HF, MA@HF, and MA@PNS-HF scaffolds in PBS solution at 37 °C for 14 days. [file research.0138.f1.docx]

**Supplementary information for**

**Living Chinese Herbal Scaffolds from Microfluidic Bioprinting for Wound Healing**

Xiaocheng Wang^1,2^, Jinxuan Jia^2^, Mengying Niu^2^, Wenzhao Li^2^, Yuanjin Zhao^1,2,3,4,^*

^1^ Department of Rheumatology and Immunology, Nanjing Drum Tower Hospital, School of Biological Science and Medical Engineering, Southeast University, Nanjing, 210096, China

^2^ Oujiang Laboratory (Zhejiang Lab for Regenerative Medicine, Vision and Brain Health), Wenzhou Institute, University of Chinese Academy of Sciences, Wenzhou, Zhejiang 325001, China

^3^ State Key Laboratory of Bioelectronics, School of Biological Science and Medical Engineering, Southeast University, Nanjing 210096, China

^4^ Chemistry and Biomedicine Innovation Center, Nanjing University, Nanjing 210023, China

* Email: [yjzhao@seu.edu.cn](mailto:yjzhao@seu.edu.cn)


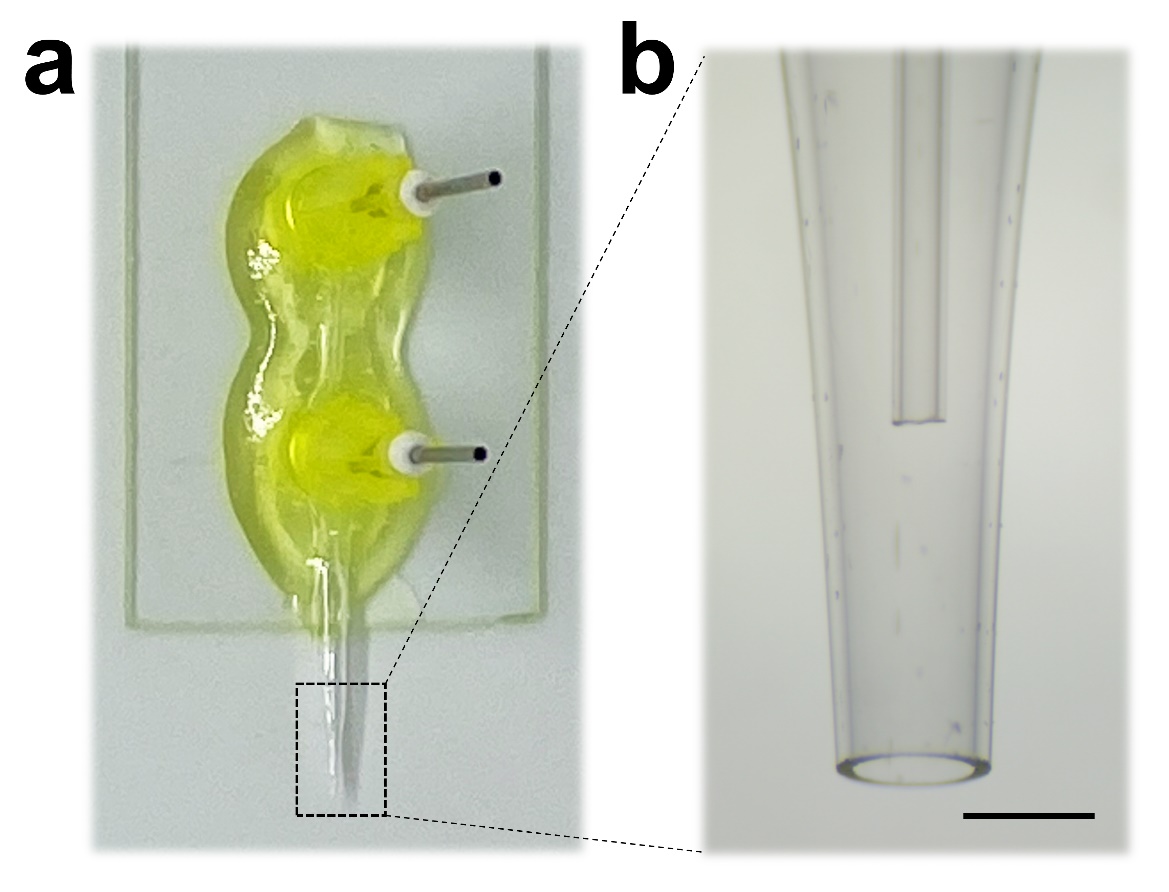


**Fig. S1.** (a) Photograph and (b) optical image of the custom-made coaxial capillary microfluidic chip with a spindle capillary (orifice diameter: 150 μm) and a tapered capillary (orifice diameter: 480 μm). Scale bar, 500 μm.


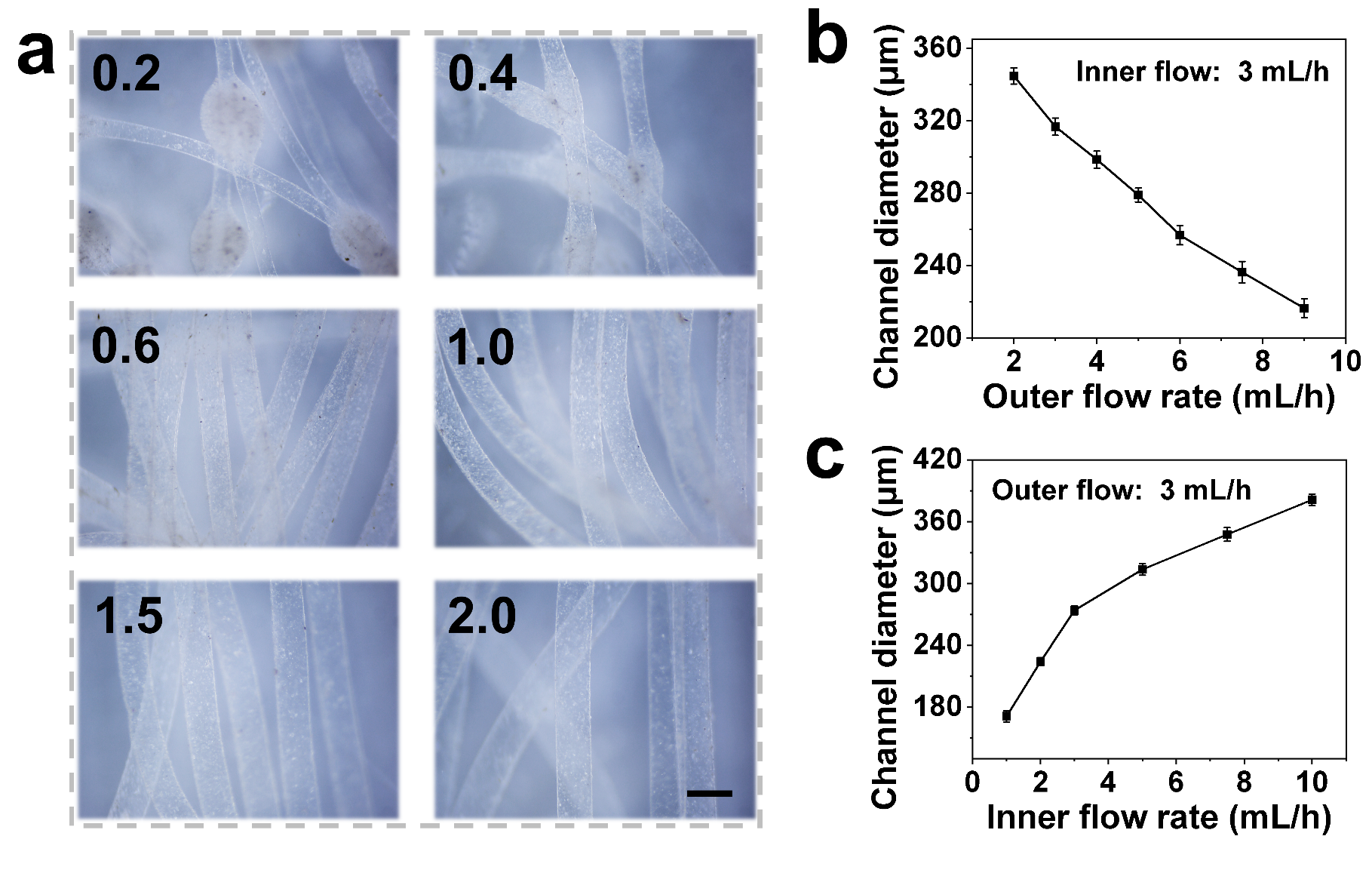


**Fig. S2.** (a) Photographs of the generated hollow microfibers from microfluidic spinning at the varied inner-to-outer flow ratios from 0.2 to 2.0. Scale bar, 500 μm. (b-c) Relationships between the channel diameter of the hollow microfibers and the flow rates of the outer (b) or inner (c) phases.


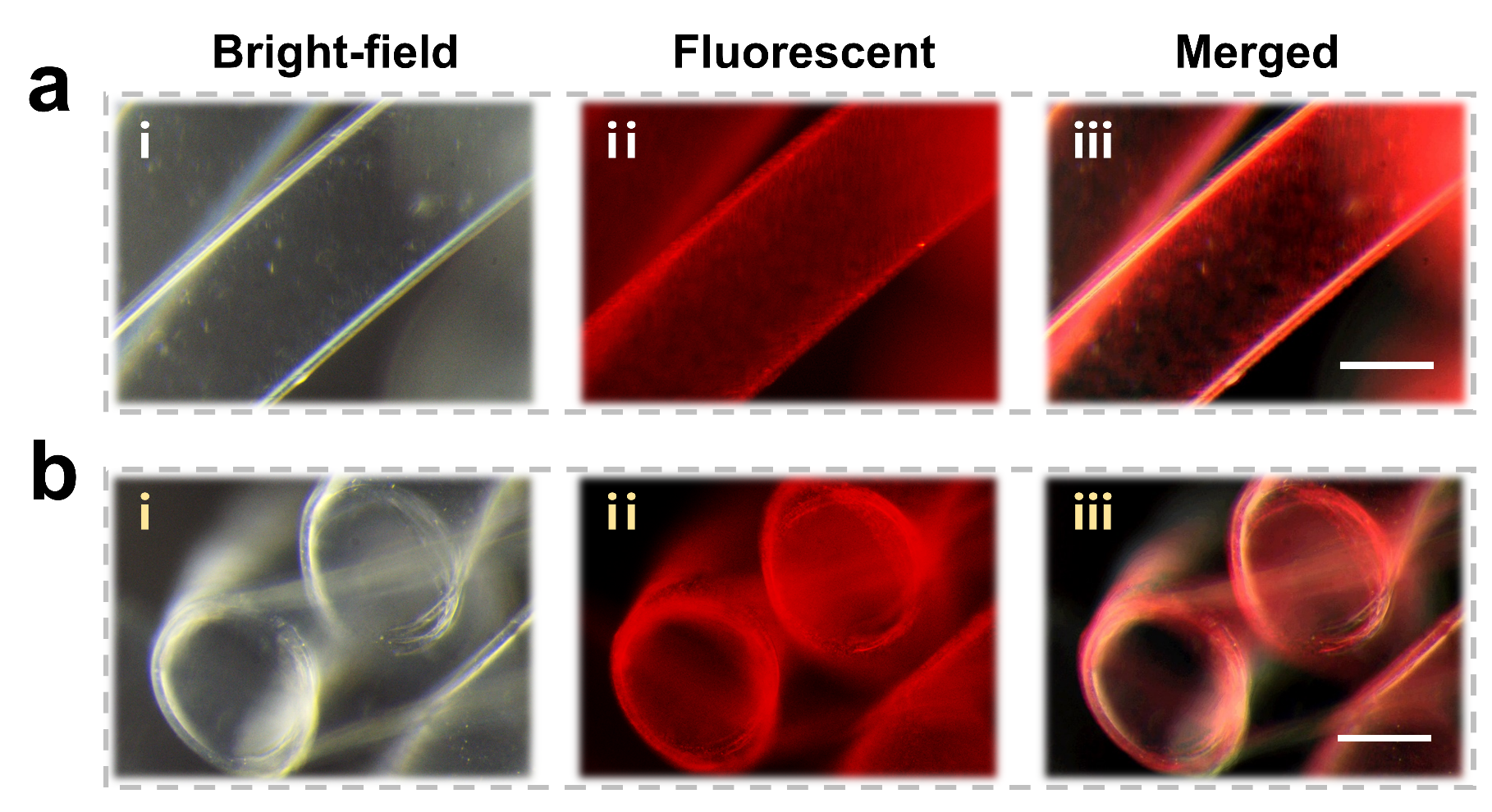


**Fig. S3.** (a) Top and (b) section views of optical and fluorescent micrographs of the hollow microfibers. Scale bars, 200 μm.


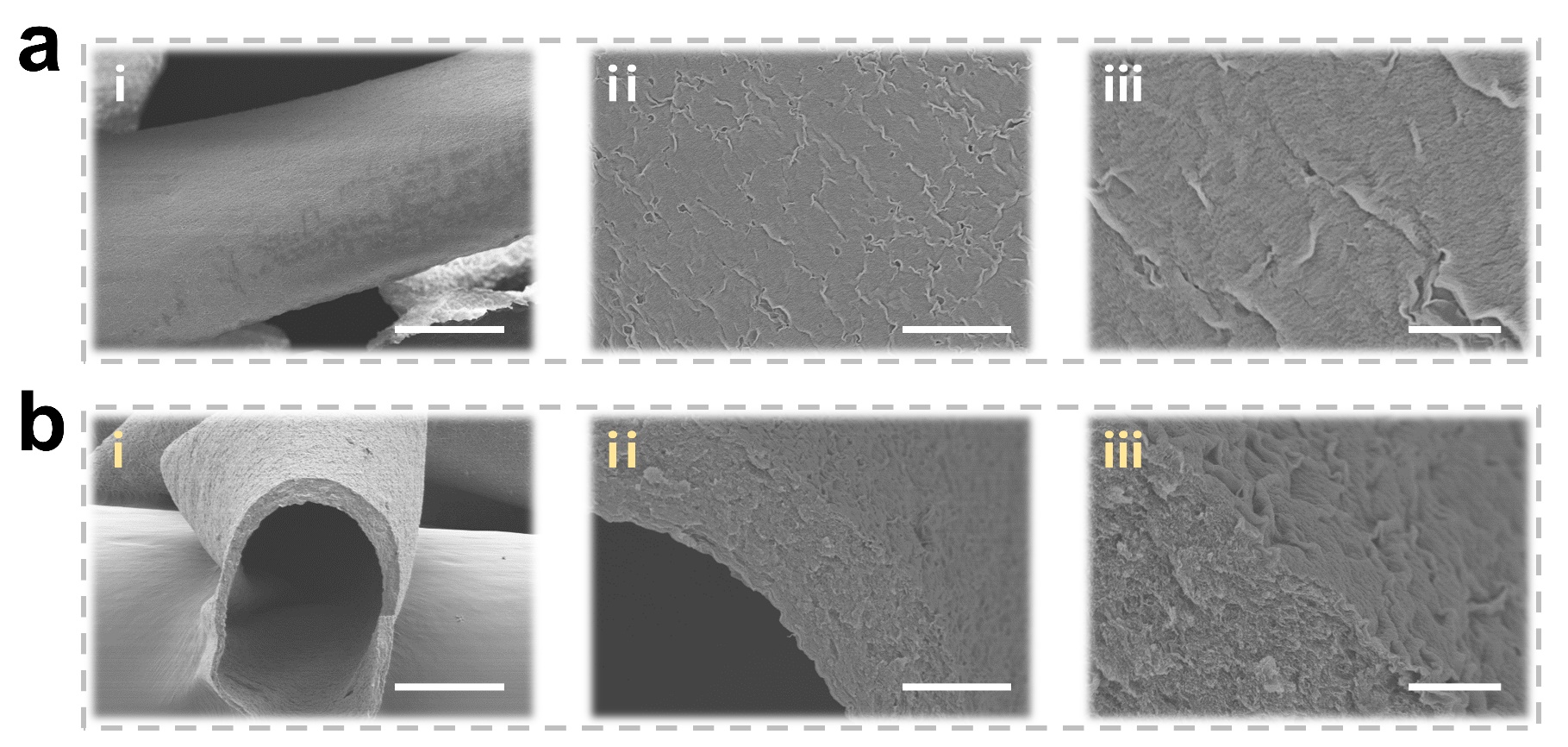


**Fig. S4.** (a) Top and (b) sectional views of SEM morphologies of the hollow microfibers at different magnifications. Scale bars indicate 100 μm in (ⅰ), 20 μm in (ⅱ), and 5 μm in (ⅲ).


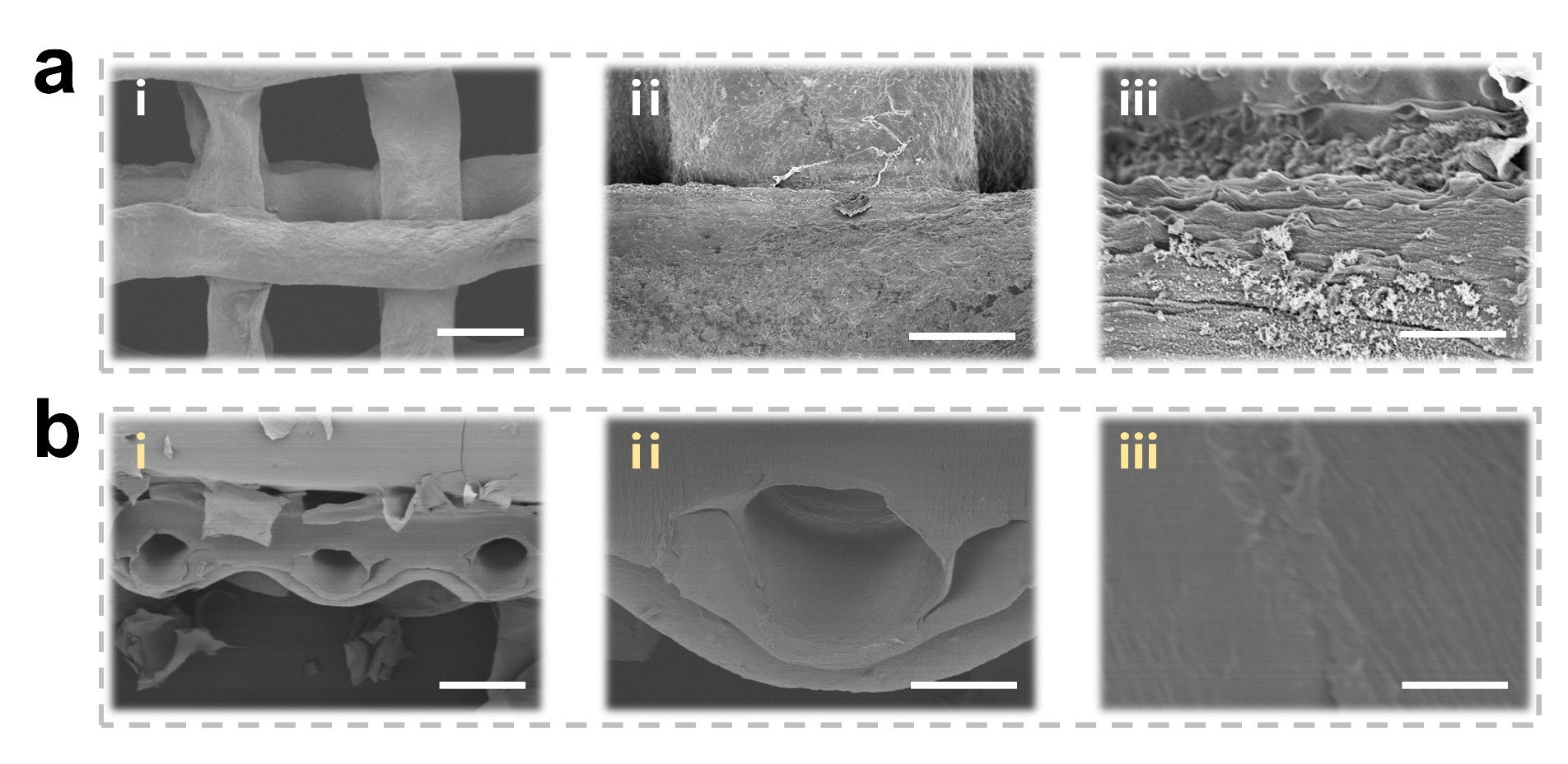


**Fig. S5.** (a) Top and (b) sectional views of SEM morphologies of the HF scaffolds without PNS loading at different magnifications. Scale bars: 300 μm in (ⅰ), 100 μm in (ⅱ), and 10 μm in (ⅲ).


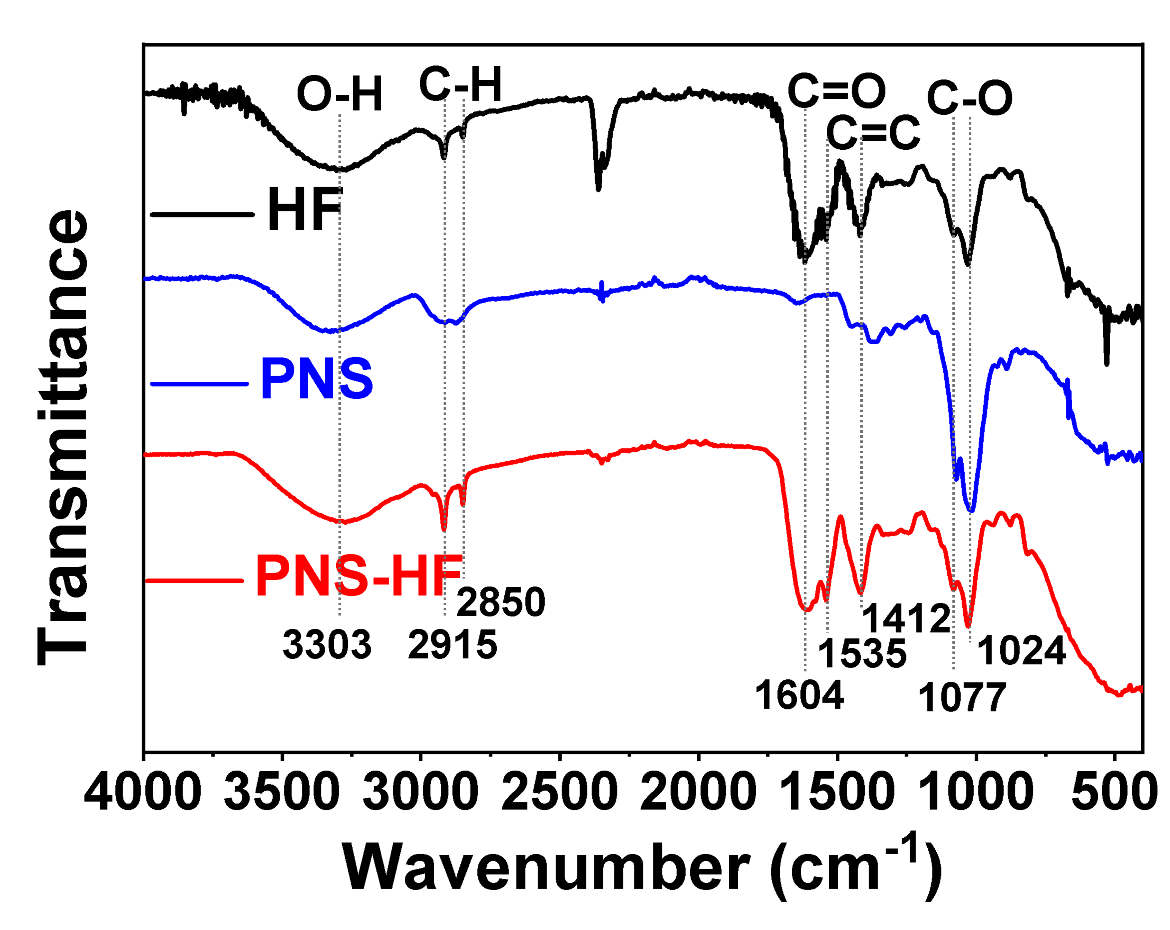


**Fig. S6**. FTIR spectra of the PNS powder, HF and PNS-HF scaffolds.


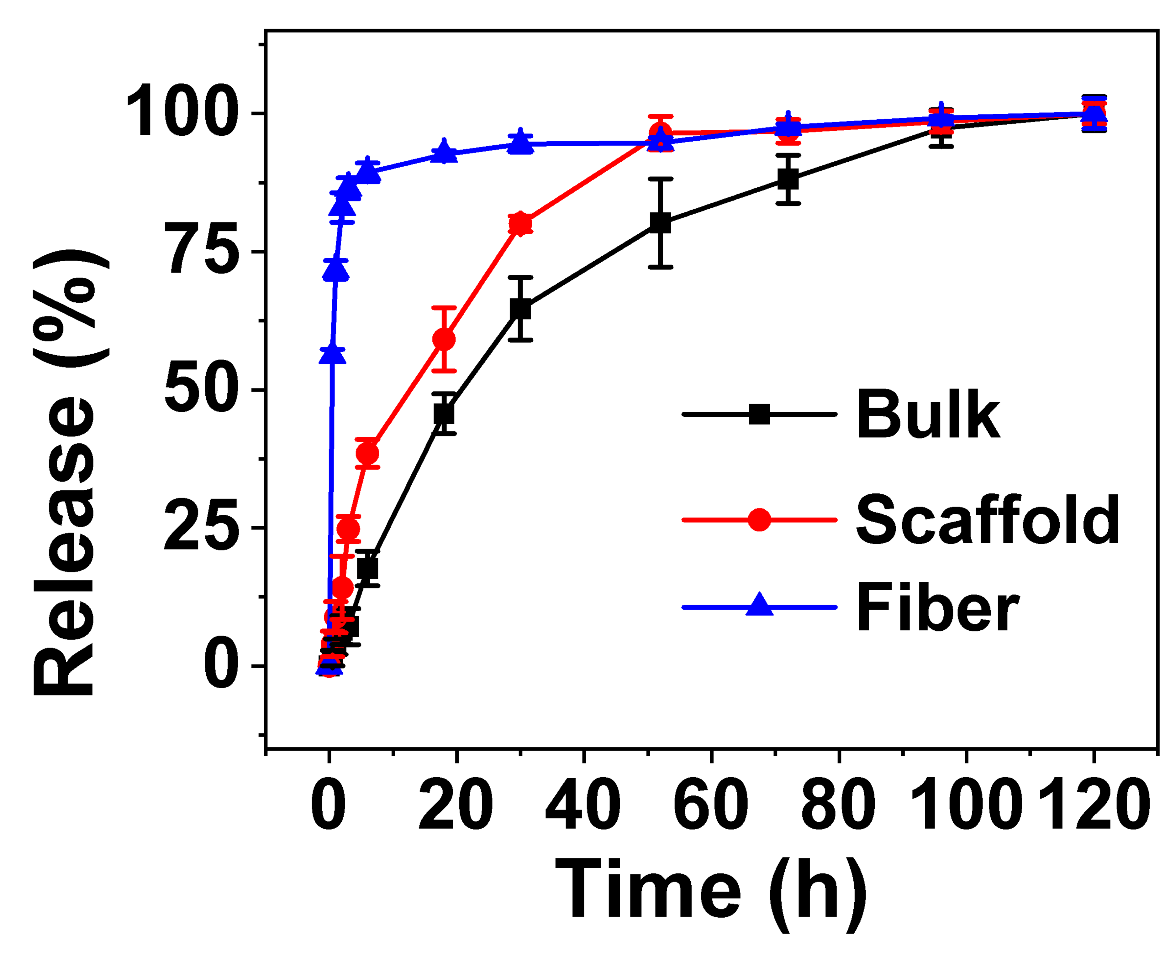


**Fig. S7**. PNS release profiles of the PNS-laden hydrogel bulks, hollow microfibers and scaffolds (PNS concentration: 25 μg/mL) in PBS at 37 ℃.


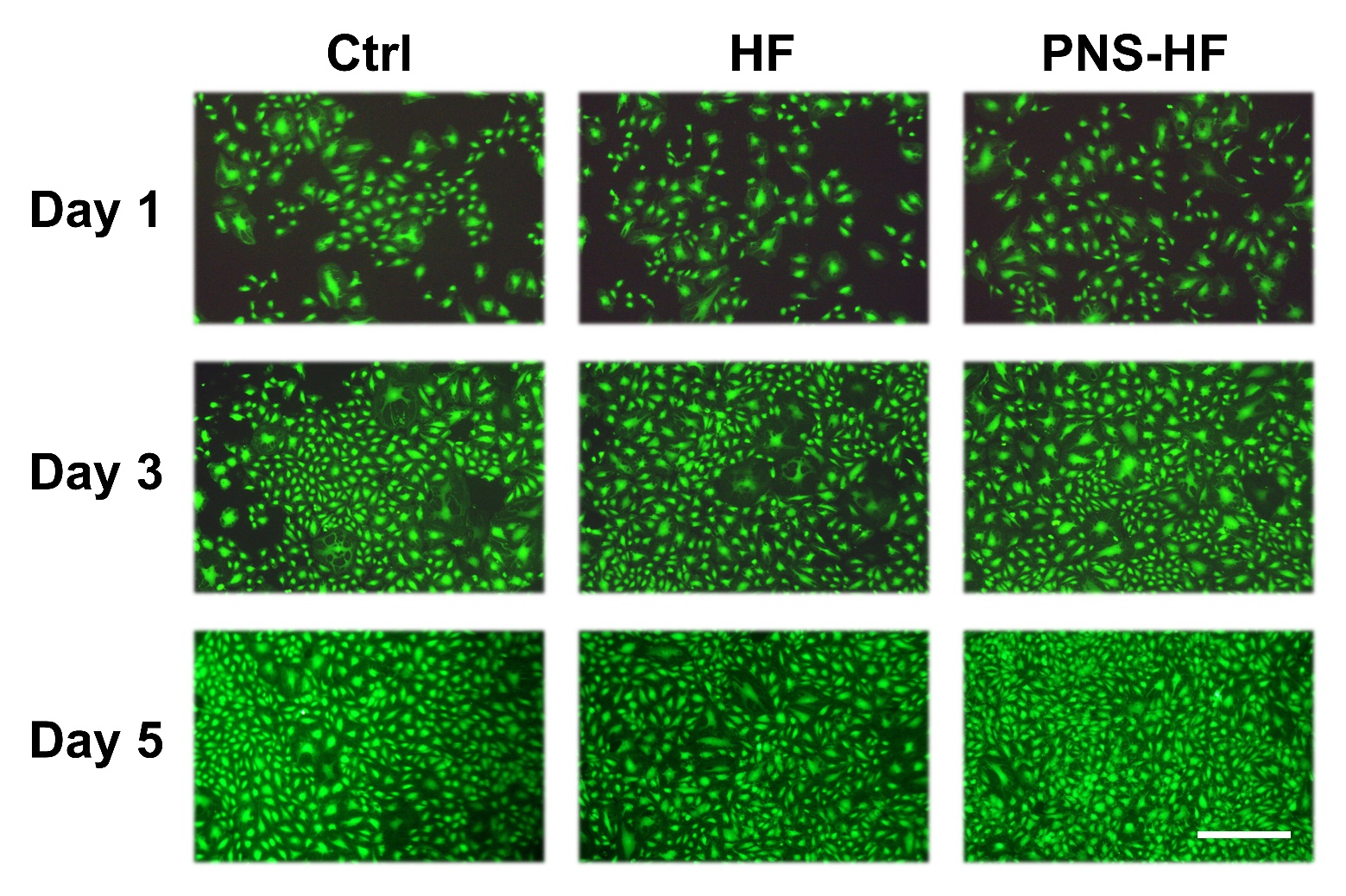


**Fig. S8**. Live/dead staining of HUVECs cultured with HF and PNS-HF scaffolds. Alive or dead cells were in green or red, respectively. Scale bar, 500 μm.


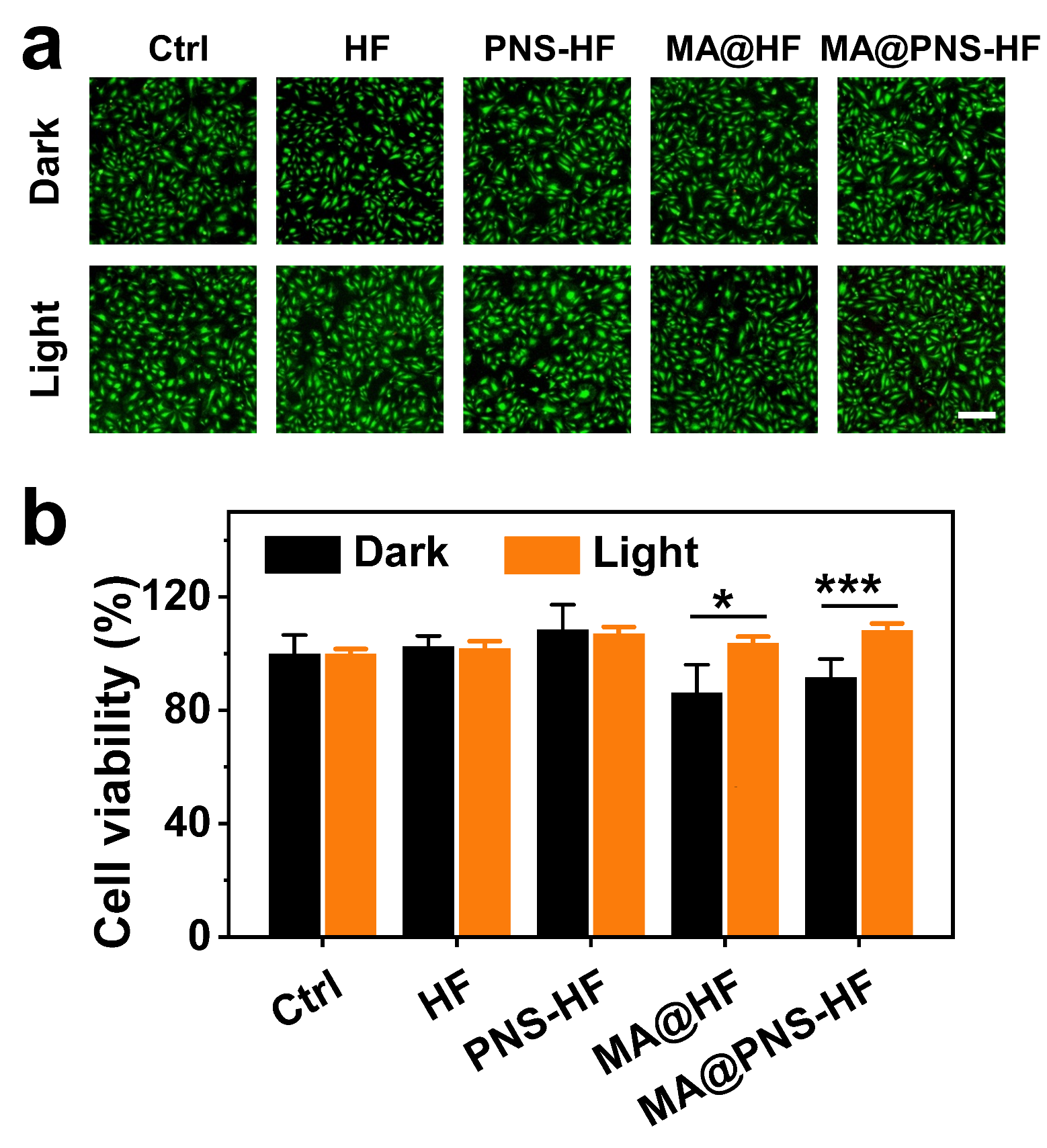


**Fig. S9**. (a) Live/dead staining of HUVECs cultured with HF, PNS-HF, MA@HF and MA@PNS-HF scaffolds for 3 days under 1% oxygen conditions. Scale bar, 500 μm. (b) Cell viability of the hypoxic HUVECs cultured with different scaffolds for 3 days.


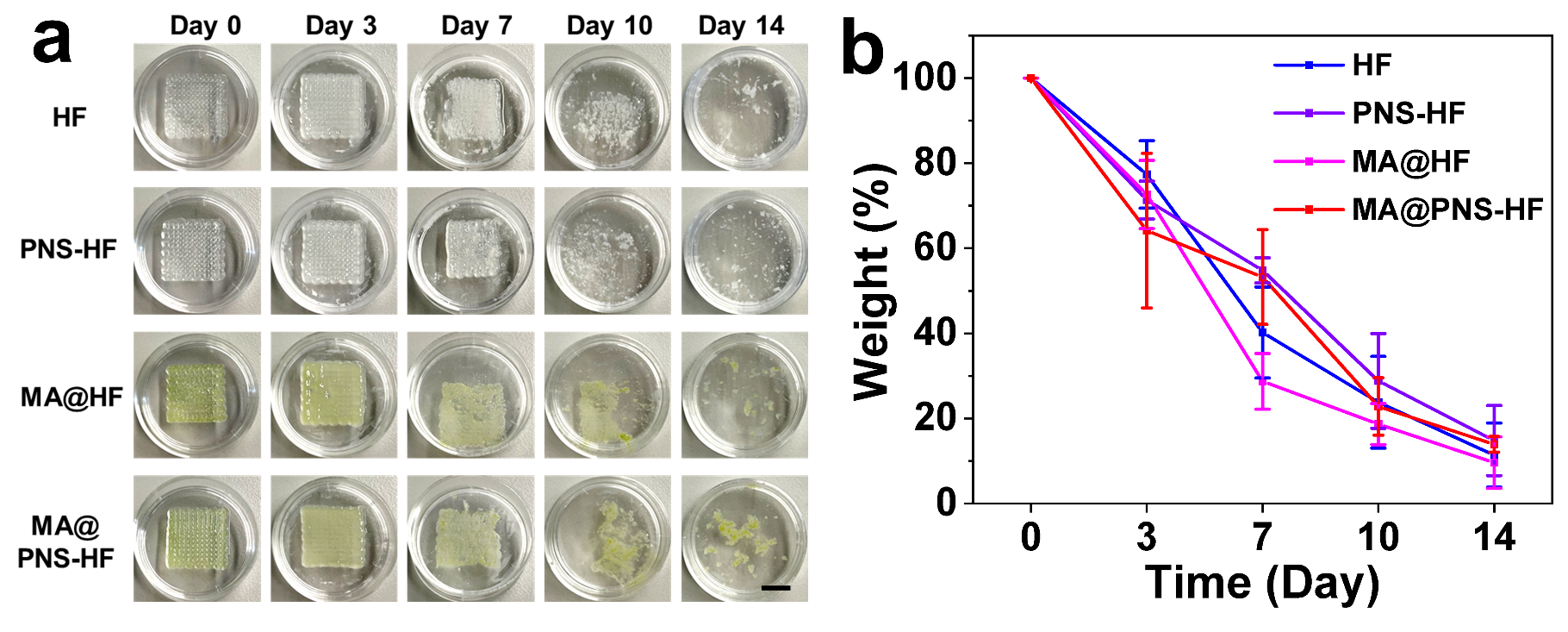


**Fig. S10**. (a) Representative photographs of HF, PNS-HF, MA@HF and MA@PNS-HF scaffolds immersed in PBS solution at 37 ℃ for 14 days. Scale bar, 1 cm. (b) Semi-quantitative analysis of the residual scaffold weight at different time point points.
